# The use of coping strategies “shift-persist” mediates associations between physical activity and mental health problems in adolescents: a cross-sectional study

**DOI:** 10.1186/s12889-021-11158-0

**Published:** 2021-06-10

**Authors:** Johan Dahlstrand, Peter Friberg, Jonatan Fridolfsson, Mats Börjesson, Daniel Arvidsson, Örjan Ekblom, Yun Chen

**Affiliations:** 1 School of Public Health and Community Medicine, Sahlgrenska Academy, University of Gothenburg, Gothenburg, Sweden; 2 The Swedish Institute for Global Health Transformation (SIGHT), Royal Swedish Academy of Sciences, Stockholm, Sweden; 3 Center for Health and Performance, Department of Food and Nutrition, and Sport Science, Faculty of Education, University of Gothenburg, Gothenburg, Sweden; 4 Center for Health and Performance, Department of Molecular and Clinical Medicine, Sahlgrenska Academy, University of Gothenburg, Gothenburg, Sweden; 5 Department of MGA, Sahlgrenska University Hopsital, Region Västra Götaland, Gothenburg, Sweden; 6 Department of Physical Activity and Health, Swedish School of Sport and Health Sciences, Stockholm, Sweden

**Keywords:** Adolescent, Stress, Psychosomatic symptoms, Coping strategies, Accelerometer, Physical activity, Sedentary time, Leisure time

## Abstract

**Background:**

Self-perceived mental health problems among adolescents has had an upward trend. Concurrently, adolescents’ physical activity (PA) has been falling whilst sedentary time (SED) has increased. There is a lack of research using accelerometer measured PA and SED to study their relationships to perceived stress and psychosomatic symptoms, both frequently observed mental health problems among adolescents. Whether coping strategies is one of the mechanisms underlying such relationship is less clear.

**Methods:**

A total of 2283 13-year olds were enrolled in the baseline examination of the STARS (STudy of Adolescence Resilience and Stress) study in Western Sweden. Light-, moderate-, vigorous-intensity PA (LPA, MPA and VPA) and SED were measured using hip-worn ActiGraph GT3X+ accelerometer. A total of 1284 adolescents provided valid accelerometer data (at least 4 days with ≥10 h per day). PA and SED during school-time and leisure-time were analysed separately. Surveys were utilized to monitor perceived stress, psychosomatic symptoms and the use of coping strategies “shift-persist”. Logistic regression and mediation analyses were performed adjusting for gender, ethnicity, socioeconomic status and puberty development.

**Results:**

We observed that more time spent in PA was associated with less stress in adolescents. The associations were observed for LPA (Odds ratio for LPA per 60 min: 0.557 (95% CI 0.399–0.776), VPA (Odds ratio for VPA per 15 min: 0.688 (95% CI 0.588–0.806) and MVPA (Odds ratio for MVPA per 15 min: 0.795 (95%CI 0.718–0.879) during leisure time, but not during school time. Similar associations were observed between leisure time PA and psychosomatic symptoms. The associations remained statistically significant even after adjusting for the confounders. Further, our data showed that adolescents who engaged more time in PA during leisure time were more likely to adopt the coping strategies of “shift-persist”. Mediation analysis showed that the use of “shift-persist” mediated the associations between leisure time PA and stress/psychosomatic symptoms.

**Conclusions:**

Leisure time physical activity, irrespective of intensity, may facilitate successful coping with stress and stress-related mental health problems in adolescents.

**Supplementary Information:**

The online version contains supplementary material available at 10.1186/s12889-021-11158-0.

## Introduction

Mental health problems affect 10–20% of children and adolescents worldwide [1] and are the leading causes of disability in young people aged 10–24 years [2]. Mental health problems commonly have their onset during adolescence [3], implying that it is important to identify modifiable risk factors in order to prevent the development of mental health problems in adolescents and in later life. In Sweden, the proportion of 13- and 15-year-old students reporting multiple psychosomatic symptoms, such as headaches and difficulty in sleep, has doubled since the mid-1980s [4], and psychosomatic symptoms are associated with increased odds ratios for functional impairment in adolescents’ everyday life [5]. There is a marked increase in stress starting from age of 13 years and with a substantial gender gap, i.e. girls reported higher level of stress than boys [6]. Further, somatic symptoms in adolescence predict severe adult mental illness measured by hospital-based care [7].

Coinciding with the increased prevalence of stress and psychosomatic symptoms, there is accumulating evidence showing that the level of physical activity in children and adolescents is decreasing. A recent review based on longitudinal studies with accelerometer measured moderate-to-vigorous physical activity (MVPA) across childhood to adolescence shows that there is a significant annual decline in MVPA across all age groups [8].

A growing body of research provides evidence for a positive association between physical activity and mental health outcomes in adolescents. Depression is mostly studied, while stress and negative affect are much less studied (for review, see [9, 10]). It remains unclear whether physical activity benefits adolescents’ overall mental well-being or whether the benefits are restricted to a certain type of mental health problems.

The majority of studies about physical activity and mental health among adolescents are based on subjective measures of physical activity [9]. Accelerometers have become a common tool for measuring physical activity in recent studies and provide more accurate measures of physical activity. However, accelerometer measured physical activity and sedentary time are not often used in mental health research.

Accelerometer measures of physical activity have traditionally been categorized into sedentary time (SED), light-, moderate- and vigorous-intensity physical activity (LPA, MPA and VPA), where the last two categories often have been merged into MVPA. Studies and guidelines about the effects of physical activity on general health as well as mental health have been focused on MVPA. The role of sedentary behaviour/time and LPA on adolescents’ mental health is less clear and results are controversial. SED has been shown to increase in children and adolescents [11, 12]. The decrease in total physical activity between 12 and 16 years of age has been shown to be driven by both decreasing LPA and increasing SED [13]. Increased screen time for leisure, a commonly used proxy measure for SED, has been shown to be associated with depressive symptoms among adolescents (for review, see [14]). In one study, self-reported screen time was shown to be positively associated with mental distress in adolescents, whereas accelerometer measured SED was not [15]. Another study showed that LPA was associated with a reduction in depressive symptoms in adolescents [13]. Other negative psychological outcomes such as stress and psychosomatic symptoms, more frequently observed among adolescents, have not been studied.

While increased SED and decreased physical activity have been frequently reported, there are also reports showing that in Sweden, the proportion of adolescents who reported leisure time exercise (corresponding to VPA) at least 4 times a week has increased since 1985/86 [16]. Adolescents’ physical activity and sedentary behaviours vary among different segments of the day and week [17]. Breaking the day/week into smaller time segments could be informative for future physical activity interventions in adolescents. We know little about the relationship between adolescents’ mental health problems and SED/physical activity during school- and leisure-time, respectively.

The mechanisms underlying the associations between physical activity and adolescent mental health are less clear. The behavioural mechanism hypothesis proposes that changes in mental health outcomes resulting from physical activity are mediated by changes in relevant and associated behaviours, such as sleep volume and quality, coping and self-regulation skills [18]. Coping, i.e. strategies for how to handle stressful situations, and especially cognitive strategies, like thinking and reasoning, is important for mental health. Under stress, a more beneficial coping style may confer resilience and reduce the activation of the physiological systems. Coping strategies “shift-persist” consist of reframing appraisals of current stressors more positively (shifting), while simultaneusly persisting with a focus on the future, and have been shown to play an important role in adolescents’ mental health [19]. Few studies have examined coping strategies [18], and mediation models are needed to identify the mechanisms responsible for any changes in mental health resulting from physical activity and sedentary behaviour [9].

In view of the above, the present study aimed to:

1. monitor physical activity and SED during school- and leisure-time among adolescents with accelerometers;
2. investigate whether physical activity and SED were associated with stress and psychosomatic symptoms in adolescents; and if so,
3. examine whether the use of coping strategies “shift-persist” mediated the association.

## Methods

### Participants

We used data from the STARS (Study of Adolescence Resilience and Stress) study, an on-going prospective and observational cohort study initiated in autumn 2015. Data used in this study were from the baseline examinations during autumn 2015 until spring 2019. Participants were 7th grade pupils recruited from 54 schools in 16 municipalities in Western Sweden. Schools were purposefully selected from different areas with various socioeconomic contexts. A total of 5084 pupils were informed and invited, and 2283 pupils entered the study. The study was approved by the Ethics Committee at the Sahlgrenska Academy at the University of Gothenburg. Both pupils and their parents or guardians gave a written informed consent to participation and use of data for research. Once the signed consent forms were received, adolescents were scheduled for baseline survey and physical examinations which were taken at the schools. They received accelerometer during the visit.

### Mental health problems

The 10-item version of the perceived stress scale (PSS-10) [20] was used to measure stress perceived by adolescents. PSS-10 was designed to measure how unpredictable, uncontrollable, and overloading respondents find their lives. Adolescents were asked to rate each statement with a 5-point frequency scale that ranges from 0 (never) to 4 (very often). A maximum of one missing item was accepted and missing item was replaced by intrapersonal mean. The total score ranged from 0 to 40, with higher score representing higher level of stress.

The psychosomatic problem scale was used to measure psychosomatic symptoms including difficulty in concentrating and sleeping, suffering from headaches and stomach-aches, poor appetite, feeling tense, low and dizzy [6]. Participants were asked to choose between 0 (never) and 4 (always). A maximum of one missing item was accepted and missing item was replaced by intrapersonal mean before a total score was calculated. The total score ranged from 0 to 32, with higher score representing higher level of psychosomatic symptoms.

In our samples, the Cronbach’s alpha coefficients for the PSS-10 scale (α = 0.812) and psychosomatic problem scale (α = 0.837) indicated high internal consistency reliability of these scales. The item-total correlations are 0.491 ± 0.082 for PSS-10 scale and 0.566 ± 0.030 for psychosomatic problem scale. Thus our way of handling item-level missing data is acceptable according to Graham [21]. We categorized the level of stress and psychosomatic symptoms into quartiles and defined lowest through highest quartiles as low, medium low, medium high and high.

### Measures of physical activity

Adolescents were asked to wear an accelerometer (ActiGraph GT3X+) over the hip during their waking hours for 7 days, except during water activities. Raw accelerometer data was processed to ActiGraph counts [22]. Consecutive periods of ≥60 min of zero counts, with allowance of up to 2 min with maximum 100 counts per minute, were defined as non-wear time. We applied wear time requirement of ≥10 h per day and ≥ 4 days per week to constitute a valid measurement. We included those with no weekend day. School time wearing was defined as weekdays during 8 am and 4 pm. Leisure time wearing was defined as weekdays from 7 am to 8 am and from 4 pm to 11 pm, plus weekends from 7 am to 11 pm. An epoch length of 3 s was used. SED, LPA, MPA and VPA were defined using Evenson’s cut points 0–100, 101–2295, 2296–4011 and > 4012 counts per minute, respectively, only considering counts from the vertical axis [23]. Evenson’s cut points have been shown to provide acceptable classification accuracy for all four levels of physical activity intensity in children and adolescents [24]. The minutes spent in different physical activity intensities were calculated.

### Potential mediating variable

The coping strategies “shift-persist” were measured as previously described [19]. The tendency to shift oneself in response to stressors was measured using the Positive Thinking scale of the Responses to Stress questionnaire, while the Life Orientation Test was used to measure future persistence. In our samples, the Cronbach’s alpha coefficients for the “shift-persist” questionnaires is 0.722. To create a total “shift-persist” score, responses to the shift and persist measures were first standardized (because they are on different scales), and then summed. Higher scores indicate using a higher combination of the “shift-persist” strategies.

### Potential confounders

Data on gender and migration background were collected. Family socioeconomic status (SES) was measured using “Family Affluence Scale” [25]. Puberty was determined using self-assessed Tanner staging by adolescents. Girls were given line drawings of girls showing the 5 stages of breast and pubic hair development with appropriate written descriptions. Boys were given line drawings of boys showing the 5 stages of genital and pubic hair development with appropriate written descriptions. Each subject was asked to select the drawing and stage that best indicated his/her own development.

### Body mass index (BMI)

Height and weight were measured without shoes. Age- and sex-specific BMI z-scores were calculated and overweight and obese were identified using the Extended International BMI cut-offs [26].

### Statistical analysis

All continuous variables were tested for normal distribution by Kolmogorov-Smirnov test and visually checked using histogram and normal probability quantile-quantile plot (Q-Q plot). Student’s t-test was used for analyzing differences between two groups and ANOVA followed by Bonferroni correction was used for analyzing differences among three or more groups. Chi-square test was used for categorical variables.

The association between physical activity and mental health outcomes were evaluated using ordinal logistic regression models. We used units of 15 min for VPA and MVPA, and 60 min for SED and LPA to avoid large numbers of minutes producing very small model coefficients that are hard to interpret, as described earlier [13]. The results are expressed as odds ratio (OR) with 95% confidence interval (CI). To adjust for potential confounding, sex, family SES, migration background and pubertal stage were all included in the models.

**Fig. 1:**
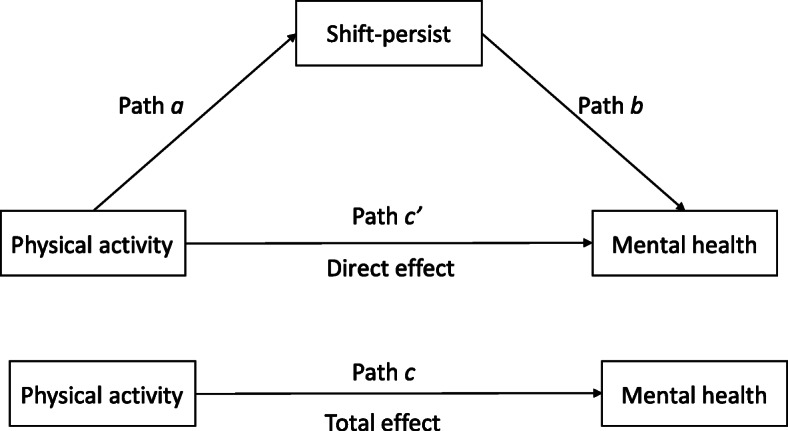
Overview of the mediation analysis

To explore the hypothesized mediating role of “shift-persist”, mediation analysis was performed using PROCESS macro (version 3.5, model 4) for SPSS, which uses a regression-based approach to mediation [27]. This macro analyzes the significance of total effect (path *c*), direct effect (path *c*’) and indirect effects (through path *a* and path *b*) as illustrated in Fig. 1 and uses bootstrapping to test the indirect effect (path *ab*) to determine if it is different from zero. Bootstrapping does not assume that indirect effect’s distribution is normal and therefore is preferable. We used 10,000 bootstrap samples for percentile bootstrap confidence intervals. To deal with heteroscedasticity we observed in some regressions, a heteroscedasticity consistent standard error and covariance matrix estimator was used for all mediation analyses.

All statistical analyses were performed with IBM SPSS 26. A two-sided *p* ≤ 0.05 was considered as statistically significant.

## Results

### Characteristics of the study sample

A total of 1756 adolescents agreed to wear accelerometer. Of these, 1284 had valid accelerometer data, i.e. ≥10 h per day and ≥ 4 days per week. Among adolescents with valid accelerometer data, there were less males, more with Swedish background and less with overweight/obesity than those without valid accelerometer data (including those who did not wear accelerometer). There was no difference in SES between the two groups. The level of stress and psychosomatic symptoms were slightly but significantly lower in adolescents with valid accelerometer data than those without (Table 1).

**Table 1:** Description of STARS participants with or without valid accelerometer data

	With (*n* = 1284)	Without (*n* = 999)	*P*
Age (year)	13.6 ± 0.4	13.6 ± 0.4	ns
Male	41.4%	48.4%	< 0.001
Family SES			ns
Low	25.8%	28.9%	
Medium	59.6%	57.9%	
High	14.6%	13.2%	
Living with both parents	74.6%	72.7%	ns
Swedish background (%)	82.6%	74.3%	< 0.001
BMI z-score	0.26 ± 0.96	0.32 ± 1.00	ns
Overweight/Obesity (%)	14.5%	17.5%	0.031
Stress	15.3 ± 6.2	15.9 ± 6.1	0.022
Q1 low	0–11	0–11	
Q2 medium low	12–15	12–15	
Q3 medium high	16–20	16–20	
Q4 high	21–36	21–36	
Psychosomatic symptoms	11.4 ± 5.4	12.0 ± 5.9	0.012
Q1 low	0–8	0–8	
Q2 medium low	9–11	9–11	
Q3 medium high	12–15	12–15	
Q4 high	16–32	16–32	

*BMI* Body mass index, *SES* Socioeconomic status, *ns* Not significant

### The level of physical activity

Girls had higher level of SED and lower levels of LPA, MPA, VPA and MVPA than boys (Table 2). When dividing into school and leisure time, all but SED, LPA and MPA during leisure time were statistically significant different between sexes.

**Table 2:** Sedentary time and time spent in different physical activity intensities in 13-year olds measured by accelerometer

	Male (***n*** = 531)	Female (***n*** = 753)	***P***
**Valid days**	6.0 ± 1.6	5.9 ± 1.4	0.391
**Total**			
SED (min/d)	769.0 ± 42.0	789.0 ± 36.4	< 0.001
LPA (min/d)	120.3 ± 24.7	107.7 ± 23.1	< 0.001
MPA (min/d)	32.7 ± 10.0	30.7 ± 8.8	< 0.001
VPA (min/d)	32.0 ± 14.8	27.0 ± 11.8	< 0.001
MVPA (min/d)	64.7 ± 21.1	57.7 ± 16.7	< 0.001
**School time**			
SED (min/d)	282.8 ± 49.7	300.6 ± 49.5	< 0.001
LPA (min/d)	57.3 ± 17.1	46.3 ± 13.5	< 0.001
MPA (min/d)	16.4 ± 6.0	15.2 ± 5.6	< 0.001
VPA (min/d)	14.9 ± 7.2	12.5 ± 6.6	< 0.001
MVPA (min/d)	31.3 ± 11.1	27.7 ± 9.7	< 0.001
**Leisure time**			
SED (min/d)	486.1 ± 57.7	488.5 ± 55.6	0.464
LPA (min/d)	61.1 ± 18.1	59.4 ± 17.6	0.094
MPA (min/d)	15.9 ± 7.4	15.2 ± 6.3	0.081
VPA (min/d)	16.8 ± 11.0	14.1 ± 8.2	< 0.001
MVPA (min/d)	32.8 ± 16.8	29.4 ± 13.0	< 0.001

*SED* Sedentary time, *LPA* Light physical activity, *MPA* Moderate physical activity, *VPA* Vigorous physical activity, *MVPA* Moderate-to-vigorous physical activity

We observed SES-related difference in leisure time MVPA, being lower in adolescents with low SES (28.7 ± 13.8 min/day) compared to medium SES (31.5 ± 15.2 min/day, *p* = 0.012) and high SES (31.4 ± 14.2 min/day, *p* = 0.151). We did not find statistically significant SES-related difference in school time MVPA.

Adolescents with immigrant background had higher level of MVPA during school time (31.5 ± 11.5 min/day) than those with Swedish background (28.7 ± 10.1 min/day, *p* < 0.001). During leisure time, however, adolescents with Swedish background had higher MVPA (31.2 ± 14.4 min/day) than those with immigrant background (28.9 ± 16.2, *p* = 0.032).

### Correlation between physical activity and mental health problems

Bivariate correlation showed that there was a statistically significant association between mental health problems and different measures of physical activity. The associations were driven by differences in physical activities during leisure time, but not school time (Table S1). We did not observe any statistically significant association between mental health problems and SED, neither during school time nor leisure time. The following analyses were therefore focused on measurements during leisure time. The strength of the relationship was small, e.g. the correlation coefficient between stress and leisure time VPA was − 0.112 (*p* < 0.001).

The fully adjusted models indicated that leisure time LPA, MPA, VPA and MVPA were negative and significant predictors for stress (Table 3). For example, every 60 min/day increase in leisure time LPA would result in a 36.6% decreased risk of being in a higher stress category after controlling for sex, SES, migration background and pubertal stage. We observed statistically significant difference between the lowest quartile and the highest quartile. Thus, for every 60-min increase on LPA, the risk of an adolescent being in high stress decreased 49.1% when compared to low stress. Another example, every 15 min/day increase in leisure time VPA would result in a 23.3% decreased risk of being in a higher stress category after controlling for sex, SES, migration background and pubertal grade. We observed statistically significant difference between the lowest quartile and the highest quartile. Thus, for every 15-min increase on VPA, the risk of an adolescent being in high stress decreased 32.3% when compared to low stress.

**Table 3:** Association between mental health problems and physical activity/SED during leisure time measured by accelerometer

	Stress			Psychosomatic symptoms		
	Unadjusted	Adjusted	Q4 vs Q1(ref)	Unadjusted	Adjusted	Q4 vs Q1(ref)
SED (per 60 min)	1.059 (0.954–1.176)	1.027 (0.925–1.142)	1.08 (0.902–1.294)	1.029 (0.928–1.142)	1.005 (0.905–1.130)	0.994 (0.838–1.178)
LPA (per 60 min)	**0.557 (0.399–0.776)**	**0.634 (0.452–0.891)**	**0.508 (0.282–0.910)**	**0.699 (0.502–0.974)**	0.824 (0.587–1.159)	0.781 (0.452–1.349)
MPA (per 15 min)	**0.712 (0.572–0.886)**	**0.787 (0.630–0.982)**	0.754 (0.515–1.105)	0.846 (0.684–1.052)	0.917 (0.733–1.144)	0.875 (0.609–1.257)
VPA (per 15 min)	**0.688 (0.588–0.806)**	**0.767 (0.654–0.900)**	**0.677 (0.508–0.900)**	**0.658 (0.560–0.771)**	**0.732 (0.622–0.862)**	**0.613 (0.466–0.808)**
MVPA (per 15 min)	**0.795 (0.718–0.879)**	**0.850 (0.766–0.942)**	**0.800 (0.667–0.958)**	**0.811 (0.733–0.898)**	**0.863 (0.778–0.958)**	**0.796 (0.671–0.946)**

In adjusted model, sex, family socioeconomic status, migration background and pubertal stage were included as confounders. Data are presented as odds ratio (95% confidence interval). Bold numbers: statistically significant associations, *p* < 0.05. Q1: lowest quartile; Q4: highest quartile. *SED* Sedentary time, *LPA* Light physical activity, *MPA* Moderate physical activity, *VPA* Vigorous physical activity, *MVPA* Moderate-to-vigorous physical activity

Similarly, we found that leisure time VPA and MVPA were negative and significant predictors for psychosomatic symptoms (Table 3).

### The role of coping strategies “shift-persist”

Bivariate correlation analysis showed that both stress and psychosomatic symptoms were negatively associated with coping strategies “shift-persist”. Further, leisure times spent in different physical activity intensities, but not SED, were found to be positively correlated with coping strategies “shift-persist” (Table S1).

To investigate whether coping strategies “shift-persist” mediate the relationship between physical activity and mental health problems, we performed mediation analyses. Results indicated that leisure time LPA, VPA and MVPA were indirectly related to mental health problems through their relationship with coping strategies “shift-persist” (Table 4). Adolescents who spent more time in LPA, VPA or MVPA during leisure time were more likely to have higher “shift-persist” score. Having higher “shift-persist” was related to lower level of stress or psychosomatic symptoms. For example, if we consider an average adolescent in our sample and keep confounders unchanged, an increase in 15 min of leisure time VPA was associated with 3.0% increase in “shift-persist” score, which in turn was associated with 12.3% decrease in stress. Another example, for an average adolescent in our sample, an increase in 60 min of leisure time LPA was associated with 3.7% increase in “shift-persist” score, which in turn was associated with 4.4% decrease in stress.

**Table 4:** Direct and indirect associations (via “shift-persist”) between leisure time physical activity and mental health problems

	Path ***a*** B (95%CI)	Path ***b*** B (95%CI)	Indirect effect Path ***ab*** B (95%CI)^a^	Direct effect Path ***c’*** B (95% CI)	Total effect Path ***c*** B (95%CI), R^2^ for total effect model
**LPA→Stress**	0.0126*** (0.0075, 0.0178)	−0.2757*** (− 0.3044, − 0.2469)	**−0.0035 (− 0.005, − 0.002)**	−0.0008 (− 0.0038, 0.0023)	−0.0043** (− 0.0075, − 0.001), 0.075
**VPA→Stress**	0.024*** (0.0148, 0.0332)	− 0.2741*** (− 0.3029, − 0.2454)	**−0.0066 (− 0.0092, − 0.004)**	−0.0034 (− 0.0095, 0.0028)	−0.01** (− 0.0162, − 0.0038), 0.078
**MVPA→Stress**	0.0144*** (0.0085, 0.0203)	− 0.2743*** (− 0.303, − 0.2456)	**−0.0040 (− 0.0056, − 0.0023)**	−0.0022 (− 0.0061, 0.0016)	−0.0062** (− 0.0101, − 0.0022), 0.077
**LPA→PSP**	0.0125*** (0.0074, 0.0176)	− 0.2663*** (− 0.2985, − 0.2341)	**−0.0033 (− 0.0048, − 0.002)**	−0.0014 (− 0.0018, 0.0045)	−0.002 (− 0.0053, 0.0013), 0.094
**VPA→PSP**	0.024*** (0.0147, 0.0332)	−0.2597*** (− 0.2917, − 0.2276)	**−0.0062 (− 0.0087, − 0.0037)**	−0.0061* (− 0.0121, − 0.002)	−0.0123*** (− 0.0185, − 0.0062), 0.104
**MVPA→PSP**	0.0143*** (0.0084, 0.0202)	−0.2612*** (− 0.2942, − 0.2301)	**−0.0038 (− 0.0054, − 0.0022)**	−0.0021 (− 0.0058, 0.0017)	−0.0058** (− 0.0097, − 0.0019), 0.099

Path *a* represents the association between physical activity and “shift-persist”. Path *b* represents the association between “shift-persist” and mental health. Path *c’* represents direct association between physical activity and mental health. Overview of the mediation analysis is illustrated in Fig. 1.

^a^Number of bootstrap samples for percentile bootstrap confidence intervals: 10000. Bold indicates statistically significant indirect effect. *B* Unstandardized coefficient, *CI* confidence interval, *LPA* Light physical activity, *VPA* Vigorous physical activity, *MVPA* Moderate-to-vigorous physical activity, *PSP* Psychosomatic symptoms. Associations are adjusted for sex, family socioeconomic status, migration background and pubertal stage. *p < 0.05, **p < 0.01; ***p < 0.001

A 95% percentile bootstrap confidence interval based on 10,000 bootstrap samples showed that the indirect effect was entirely under zero, indicating successful mediation. The direct effects (*c’*) were not statistically significant (exception VPA → PSP), indicating complete mediation. The R-squared for the Total Effect Model ranged from 0.075 to 0.104, thus explaining 7.5–10.4% of variance in the level of mental health problems.

## Discussion

Two main findings emerged from this study. First, accelerometer measured times spent in different physical activity intensities were negatively associated with the levels of stress and psychosomatic symptoms in 13-year olds. The associations were observed for LPA, VPA and MVPA during leisure time, but not during school time. Second, the use of coping strategies “shift-persist” mediated the associations between leisure time physical activity and stress/psychosomatic symptoms. Thus, our data suggest that leisure time physical activity may strengthen adolescents’ capacity to cope with stress and stress-related mental health problems.

Research regarding the impact of physical activity on mental health outcomes other than depression is lacking [9, 10]. Using accelerometer measured physical activity, we found that more time spent in physical activity was associated with less stress and psychosomatic symptoms in a healthy adolescent population. This is in line with the few existing studies showing that adolescents who reported greater physical activity also reported less stress [28, 29] as well as less somatic and psychological complaints [30]. Interestingly, we found that not only the traditional VPA and MVPA but also LPA were associated with stress and psychosomatic symptoms. Recently, LPA was shown to be associated with a reduction in depressive symptoms in adolescents [13]. LPA has been shown to have benefits for anxiety symptoms among young people aged 12–26 years [10]. The current guidelines about the effects of physical activity on general health as well as mental health in children and adolescents focus on MVPA. Our result suggests that leisure time LPA might be also important as high-intensity physical activity in alleviating stress. This is encouraging as LPA is relatively easier to achieve compare to MVPA. LPA, together with SED, constitutes the vast majority of waking daily activity. Interventions with LPA may be beneficial for people who are unlikely to adhere to more vigorous-intensity interventions. Our study provides new evidence supporting the new *WHO 2020 guidelines on Physical activity and Sedentary Behaviour* that suggest “doing some physical activity is better than doing none” [31].

Despite increasing evidence supporting the beneficial effects of physical activity on adolescents’ mental health, the underlying mechanisms are less clear. To fill this gap, we used mediation models to investigate whether the use of coping strategies “shift-persist” can be one of the underlying mechanisms. Our data showed that adolescents who engaged more time in physical activity during leisure time were more likely to adopt coping strategies “shift-persist”. This is in line with the study in adults showing that a higher degree of physical activity was linked to a greater relative preference for the reappraisal strategy of positive reinterpretation [32]. Also, Giles et al. showed that higher habitual exercise was associated with greater cognitive control of neural information and cognitive reappraisal of emotional information [33]. Thus, a persons’ physical activity may facilitate more adaptive emotion regulation in critical contexts.

Our data showed that having higher “shift-persist” was related to less stress or psychosomatic symptoms and the use of “shift-persist” mediated the associations between physical activity and stress/psychosomatic symptoms. Our finding is in line with the notion that physical activity increases well-being through better coping with stressors, including improved stressor appraisal or specific styles of coping when faced with stressful situations [34]. There is evidence supporting that the ability to adapt to stress by shifting oneself or positive reappraisal can reduce reactivity of the hypothalamic–pituitary–adrenal axis to stressors, facilitating faster stress recovery or greater resilience, and thereby, potentially greater coping success by reappraisal [35]. Indded, children with higher levels of daytime physical activity have been shown to have lower hypothalamic-pituitary-adrenocortical axis activity in response to the Trier Social Stress test [36], although this has not been confirmed in adults [37]. Taken together, spending more time in physical activity may enhance adolescent’s ability to adaptively respond to stressors, thus minimizing risk of having high stress and psychosomatic symptoms. Further longitudinal studies are warranted.

Our data showed that the associations were observed for LPA, VPA and MVPA during leisure time, but not during school time. One explanation for the lack of association between adolescent’s mental health problems and school time physical activity can be that Swedish schools provide rather equal level of physical education. Interestingly, we observed the SES-related difference in MVPA during leisure time, but not school time.

The role of SED on adolescents’ mental health is less clear and results are controversial. Using accelerometer measured SED, we did not find any significant association between SED and stress/psychosomatic symptoms in adolescents. In line with our study, Opdal showed that accelerometer-assessed SED was not associated with mental distress in adolescents [15]. Kandola et al. showed, however, that accelerometer-assessed SED increased throughout adolescence and was associated with depressive symptoms at age 18 years [13]. The reason for this controversy is unknown. We don’t have information regarding the context in which the adolescents were sedentary, therefore the impact of social component of sedentary behaviour on stress/psychosomatic symptoms is not revealed by this study.

Strengths of this study include accelerometer based measures of physical activity and SED, large population-based adolescence sample, and the use of moderation models to investigate the underlying mechanisms. Our study also has limitations. Our study was based on cross-sectional data, and did not allow conclusion on causal relation. There is evidence that relationship between sport involvement and mental health in adolescence can be bi-directional [38]. Accelerometers cannot accurately record certain activities, such as water-based activities and activities that only use the lower body. We did not apply at least one weekend day as inclusion criteria for accelerometer data as a total of 231 (18%) did not have accelerometer data for weekend. However, including the number of days of weekend wearing as an additional co-variable did not change the results from regression or mediation analysis.

## Conclusions

Our study showed that more time spent in physical activity, irrespective of intensity, is associated with less stress and psychosomatic symptoms in adolescents. In particular, the involvement of adopting coping strategies “shift-persist” in this association signals a potential mechanism through which physical activity may exert positive effects on stress management. Thus, based on our cross-sectional data, we conclude that leisure time physical activity may facilitate successful coping with stress and stress-related mental health problems in adolescents. The importance of LPA should be highlighted.

## Supplementary Information

**Additional file 1: Table S1.** Bivariate correlations between variables.

## Data Availability

All data are stored by the research group of the authors at University of Gothenburg, Sweden. Currently, only the research team has access to data. Researchers who are interested in collaboration should contact the principal investigator Professor Peter Friberg (peter.friberg@mednet.gu.se) or co-investigator Associate Professor Yun Chen (yun.chen@wlab.gu.se).

## Abbreviations

BMI: Body mass index
LPA: Light-intensity physical activity
MPA: Moderate-intensity physical activity
MVPA: Moderate-to-vigorous-intensity physical activity
VPA: Vigorous-intensity physical activity
OR: Odds ratio
PSP: Psychosomatic symptoms
SED: Sedentary time
STARS: The STudy of Adolescence Resilience and Stress
